# Modification of Insect and Arachnid Behaviours by Vertically Transmitted Endosymbionts: Infections as Drivers of Behavioural Change and Evolutionary Novelty

**DOI:** 10.3390/insects3010246

**Published:** 2012-02-29

**Authors:** Sara L. Goodacre, Oliver Y. Martin

**Affiliations:** 1 School of Biology, University of Nottingham, NG7 2RD, UK; 2 ETH Zurich, Experimental Ecology, Institute for Integrative Biology, Universitätsstrasse 16, CH-8092 Zurich, Switzerland; E-Mail: oliver.martin@env.ethz.ch

**Keywords:** reproductive parasite, host, arthropod, manipulation, bacteria

## Abstract

Vertically acquired, endosymbiotic bacteria such as those belonging to the Rickettsiales and the Mollicutes are known to influence the biology of their arthropod hosts in order to favour their own transmission. In this study we investigate the influence of such reproductive parasites on the behavior of their insects and arachnid hosts. We find that changes in host behavior that are associated with endosymbiont infections are not restricted to characteristics that are directly associated with reproduction. Other behavioural traits, such as those involved in intraspecific competition or in dispersal may also be affected. Such behavioural shifts are expected to influence the level of intraspecific variation and the rate at which adaptation can occur through their effects on effective population size and gene flow amongst populations. Symbionts may thus influence both levels of polymorphism within species and the rate at which diversification can occur.

## 1. The Effects of Bacterial Endosymbionts on the Biology of Their Hosts

The nature of the relationship between hosts and their parasites has been the focus of a wide range of studies (reviewed in [1]). One particular area of current interest is in the types of behavioural change that can be induced in a host following an infection. Studies of horizontally acquired nematode, trematode or protozoan parasites in a range of arthropods indicate that behavioural changes may be induced through altered levels of gene expression in the host CNS [2,3]. The precise mechanisms are not fully understood but in many instances the behavioural change is predicted to be the consequence of selection acting on the parasite to promote its own transmission. Behavioural changes are also induced in hosts following infection with vertically acquired, intracellular bacteria such as *Wolbachia, Cardinium* or *Spiroplasma*. These changes in host biology may also be driven by selection acting to promote bacterial transmission, but in this case the primary route is from mother to offspring. Horizontal transmission, whilst it undoubtedly occurs, is not the primary transmission route for these bacteria [4] and is therefore likely only to be important on an evolutionary timescale.

There are several main intracellular, primarily vertically acquired, bacterial lineages that are known to infect arthropods. These include bacteria from the *Rickettsiales* such as *Wolbachia*, and *Rickettsia*, members of the Bacteroidetes group such as *Cardinium* or *Flavobacteria*, Mollicutes such as *Spiroplasma* and *Arsenophonus*, a newly described bacterium lineage belonging to the Gamma-Proteobacteria. Each of these is inherited primarily through the female germ line and as such is always found within female reproductive tissue, but many are found within a much wider range of host tissue types and/or are present throughout the haemocoel. These bacteria act as selfish genetic elements, promoting their own transmission by increasing the reproductive success of infected females. They achieve this through a variety of mechanisms: (1) by causing cytoplasmic incompatibility (where uninfected females produce no offspring when mating with an infected male) (2) by biasing the sex ratio in favour of females *via* either male-killing (either early or late, *i.e.*, as embryos or as larvae) or through feminisation of genetically male individuals or (3) through inducing parthenogenesis [4,5]. A summary of bacterial phenotypes and examples of taxa in which these have been found is given in Table 1. Thus far only *Wolbachia* has been shown to cause all these phenotypes, whereas for *Cardinium* there is evidence for all of these phenotypes except male-killing. In contrast, the other microbes are all known to act as male-killers and *Rickettsia* can also be associated with parthenogenesis.

**Table 1:** Overview of the classic symbiont-induced phenotypes with responsible microbes and examples of known insect and arachnid host taxa in which they occur.

Phenotype		Symbiont		Host taxa (references)
**Male-killing**				
		*Wolbachia*		Lepidoptera: Nymphalidae: *Hypolimnas bolina* [30], *Acraea encedon* [33]
				Pseudoscorpiones: Chernetidae: *Cordylochernes scorpioides* [34]
				Araneae: Linyphiidae: *Oedothorax gibbosus* [35]
		*Rickettsia*		Coleoptera: Buprestidae: *Brachys tessellates* [36]
				Coleoptera: Coccinellidae: *Adalia bipunctata* [37], *Adalia decempunctata* [38]
		*Spiroplasma* * ixodetis*		Coleoptera: Coccinellidae: *Adalia bipunctata* [39], *Anisosticta novemdecimpunctata* [40], *Harmonia axyridis* [41]
				Lepidoptera: Nymphalidae: *Danaus chrysippus* [42]
		*S. poulsonii*		Diptera: Drosophilidae: *Drosophila willistoni* group [43]
		*Arsenophonus nasoniae*		Hymenoptera: Pteromelidae: *Nasonia vitripennis* [44]	
		*Flavobacterium* sp.		Coleoptera: Coccinellidae: *Coleomegilla maculata* [45], *Adonia variegata* [46]	
**Feminization**				
		*Wolbachia*:		Isopoda: Armidillidiidae: *Armadillidium nasatum* [47], *A. vulgare* [48] Lepidoptera: Pieridae *Eurema hecabe* [49]	
		*Cardinium*		Acari: Tenuipalpidae: *Brevipalpus* spp. [50,51]	
**Parthenogenesis**				
		*Wolbachia*		Hymenoptera: Pteromelidae: Trichogramma spp. [48,51], *Muscidifurax uniraptor* [53]	
				Hymenoptera: Scelionidae *Telenomus nawai* [54]	
				Hymenoptera: Encyrtidae*: Apoanagyrus diversicornis* [55]	
				Hymenoptera: Eucoilidae: *Leptopilina australis* [56]	
				Hymenoptera: Aphelinidae: *Aphytis disapidis* and *A. lingnanesis, Encarsia formosa* [57]	
		*Cardinium*		Hymenoptera: Aphelinidae: *Encarsia* ssp [58,59]	
				Hemiptera: Diaspididae: *Aspidiotus nerii* [60]	
		*Rickettsia*		Hymenoptera: Eulophidae: *Neochrysocharis Formosa* [61]	
**CI**				
		*Wolbachia*:		Coleoptera: Tenebrionidae: *Tribolium confusum* [62]	
				Diptera: Culicidae: *Culex pipiens* [63]	
		*Cardinium*		Acari: Tetranychidae: *Eotetranychus suginamensis* [64] and *Bryobia sarothamni* [65]	
				Hymenoptera: Aphelinidae: *Encarsia pergendiella* [66]	

Vertically acquired bacterial symbionts are extremely common in arthropods, with the most intensively studied bacterium in this context, *Wolbachia*, potentially infecting more than 60% of insect species [6]. Although *Wolbachia* could well represent the most widespread symbiont in insects, various surveys of insects and arachnids show that other symbiont types may also be highly prevalent [7,8,9]. Some strains, such as *Cardinium*, appear to be more common in arachnids than in insects, an observation that indicates that individual bacterial types do not infect all arthropod groups equally [10,11]. 

The wide range of hosts that can be infected with symbionts such as *Wolbachia* is matched by a correspondingly wide range of known additional effects on host physiological and fitness traits such as female fecundity and male fertility (Table 2). Although the physiological consequences of an endosymbiont infection in any given species are not always fully understood it is clear that these bacteria can interfere in a diverse range of processes and in some cases may even be essential to host survival or reproduction. For example the presence of *Wolbachia* has been shown to be necessary for host oogenesis in the hymenopteran parasitoid *Asobara tabida* [12]. Similar effects are known from associations between symbionts and their nematode hosts. Physiological effects are however not restricted to reproductive processes; symbionts may also alter host susceptibility to a range of biotic and abiotic factors (summarised in Table 2). For example, some bacteria have been shown to provide benefits to their host through conferring increased resistance to pathogens, elevating protection from parasitoids, altering susceptibility to insecticides and improving thermotolerance. These kinds of beneficial effects are perhaps not unexpected given that bacterially mediated resistance against pathogens and parasitoids is already known from studies of other types of bacteria to those that are the focus of this study. For example, the *Hamiltonella defensa* bacterial symbiont that infects the pea aphid *Acyrthosiphon pisum* can block larval development of the hymenopteran parasitoids *Aphidius ervi, A. eadyi* [13] and *Lysiphlebus fabarum* [14].

**Table 2:** Physiological effects of infection with vertically acquired, endosymbiotic bacteria in arthropod hosts.

Traits	Effects	Symbionts	Host species (references)
**Reproduction**			
Female fecundity	Infection decreases offspring number	*Wolbachia*	*Tribolium confusum* [67]
Female fecundity	Infection increases fecundity	*Cardinium*	*Metaseiulus occidentalis* [68]
“	“	*Wolbachia*	*Trichogramma bourarachae* [69]
“	“	*Wolbachia*	*Aedes albopictus* [70]
Fertilization	No participation of sperm in reproduction of infected females	*Wolbachia*	*Telenomus nawai* [54]
Microbe necessary for host oogenesis	Bacterium influences programmed cell death processes, so presence is essential for maturation of host oocytes	*Wolbachia*	Parasitoid wasp *Asobara tabida* [12]
“	Females treated with tetracycline or rifampicin have significantly reduced number of mature eggs in their ovaries	*Wolbachia*	*Drosophila paulistorum* [71]
Male fertility	Infection increases fertility	*Wolbachia*	*Tribolium confusum* [67]
Sperm competitive ability	Infection leads to reduced sperm competition success	*Wolbachia*	*Drosophila simulans* [72]
**Immunity**			
Resistance (fungus)	Infected females are more resistant to the entomopathogenic fungus *Beauveria bassiana*	*Wolbachia*	*Drosophila melanogaster* [73]
Resistance (viruses)	Infection induces resistance to Dengue virus and infected mosquitoes are less likely to transmit the disease	*Wolbachia*	*Aedes aegypti* [74]
“	Infection increases host resistance to *Drosophila* C virus (DCV)	*Wolbachia*	*Drosophila melanogaster* [74]
“	Infected individuals more resistant to mortality induced by the viruses DCV, cricket paralysis virus, Flock House virus	*Wolbachia*	*Drosophila melanogaster* [75]
“	Antiviral protection occurs in some yet not all fly line-*Wolbachia* strain combinations assessed	*Wolbachia*	*Drosophila simulans* [77]
**Fitness**			
Survival	Infection increases longevity	*Wolbachia*	*Drosophila melanogaster* [78],
			*Aedes albopictus* [70]
Nutritional mutualism	Infections required for host function, probably via provision of B vitamins missing in the diet the blood-feeding host	*Riesia* sp. (‘biologically highly derived species of *Arsenophonus*’ [79])	*Pediculus* and *Pthirus* species of lice [80,81]
“	Symbiont has essential nutritional role for the host (B vitamins)	*Wolbachia*	*Cimex lectularius* [82]
Metabolic provisioning	Benefit apparent under nutritional stress: if flies reared on poor diets, infected flies produce more eggs than uninfected flies	*Wolbachia*	*Drosophila melanogaster* [83]
Thermotolerance	When compared to uninfected population, infected population had significantly increased tolerance to heat shock that reached 40 °C	*Rickettsia*	*Bemisia tabaci* [84]
Protection against parasitoid	Infection enhances survival of individuals attacked by parasitic wasp (*i.e.*, possible defensive mutualism)	*Spiroplasma*	*Drosophila hydei* [85]
Susceptibilityto insecticides	Infected individuals more susceptible	*Rickettsia*	*Bemisia tabaci* [86]
Susceptibilityto insecticides	Infected individuals more susceptible	double infections *Rickettsia—Arsenophonus & Wolbachia—Arsenophonus*	*Bemisia tabaci* [85,86]

It is known that the speed at which phenotypic changes in the host can be effected by endosymbionts may be rapid [15] and it is also known that bacterial phenotypes have the potential to change [16]. Features of the bacterial genomes themselves may play a part in the latter, for instance mobile genetic elements and the presence of numerous phage are proposed to contribute to the rate at which the phenotype that is induced in the host changes from one state to another, for example from pathenogenicity to protective mutualism [13]. There is clearly, however, also a role for the interaction between host and bacterial genomes in determining phenotype because the same type of symbiont can cause different effects in different hosts. As an example, *Spiroplasma* causes male-killing in several *Drosophila* species, yet other spiroplasmas, such as that which infect the pea aphid, *Acyrthosiphon pisum* [17], are not male-killers. In fact, even the same bacterial strain need not always have the same effects. This is illustrated in Table 3, which lists a range of different phenotypes exerted by identical *Wolbachia* strains infecting different hosts. A single infecting strain may even cause different phenotypes depending upon its precise location within the host. For example the effect of the same infecting *Rickettsia* strain in whiteflies is shown to be dependent upon whether it is found widely throughout the host’s hemocoel or if it is restricted to within bacteriocytes [18]. 

**Table 3:** *Wolbachia* strains known to cause different phenotypes or effects in different hosts.

Taxonomy, references	Host species	Phenotype induced
Diptera: Drosophilidae [16]	*Drosophila recens *(natural host)	CI
	*Drosophila subquinaria *(introgressed)	Male-killing (in some host strains)
Lepidoptera: Pyralidae [88]	*Cadra cautella *(natural host)	CI (partial)
	*Ephestia kuehniella *(transinfected)	Male-killing
Isopoda: Philosciidae, Armadillidiidae and Onscidae [89]	(a) *Chaetophiloscia elongata* (natural host)	Feminization
	(a) *Armadillidium vulgare* (transinfected)	No feminization of males
	(b) *Armadillidium vulgare *(natural host)	Feminization
	(b) *Armadillidium nasatum *(transinfected)	Feminization
	(b) *Oniscus asellus* (transinfected)	No feminization of males
Coleoptera: Tenebrionidae [90]	*Tribolium confusum *(natural host)	CI
	*Tribolium madens *(natural host)	Male killing

## 2. Endosymbionts as Direct and Indirect Sources of Behavioural Change

In addition to the physiological effects already described (Table 1,Table 2,Table 3) it is known that vertically acquired endosymbiont infections can result in altered behaviour of the infected host. A summary of the types of behavioural shifts that have been observed thus far is given in Table 4. The types of change that are documented can be grouped into ‘reproductive behaviour’ (*i.e.*, those behaviours directly involved in reproduction) and non-reproductive behaviour (*i.e.*, those not directly involved in reproduction). Reproductive behaviours that are known to be directly under the influence of endosymbiont infections include mating preference, courtship, mating duration, mating frequency and post-mating behaviours of females such as oviposition. Non-reproductive behaviours, which are less frequently reported in the literature as being influenced by endosymbiont infections than reproductive behaviours, include dispersal in a linyphiid spider [19] and competitive behaviour of larvae in *Drosophila* [20].

**Table 4:** Behavioural effects of infection with vertically acquired, endosymbiotic bacteria in arthropod hosts.

Behaviour	Effects	Symbionts		Host species (reference)
**Reproductive**				
Female mating behaviour	Pre-mating isolation via selective mate avoidance, *i.e.*, avoiding mates harboring another, incompatible symbiont variant.	*Wolbachia*		*Drosophila paulistorum* [71]
“	Females of thelytokous host strain inseminated less often than arrhenotokous (sexual) females.	*Wolbachia*		*Apoanagyrus diversicornis* [55]
“	Reproductive barrier between antibiotic-induced males and females due to nonreceptivity of females.	Unknown, but not *Wolbachia*		*Galeopsomyia fausta* [91]
Female mating behaviour (and anatomy)	Females reluctant to mate and also have ananatomical alteration: major spermathecal muscle absent	*Wolbachia*		*Muscidifurax uniraptor* [53]
Mate choice	Assortative mating dependent on genotype, infection status and combination.	*Wolbachia*		*Drosophila melanogaster* [92]
“	Males prefer real females to feminized genetic males	*Wolbachia*		*Armadillidum vulgare* [93]
“	Uninfected females prefer uninfected males	*Wolbachia*		*Tetranychus urticae* [21]
Male-male competition	Infected males are more competitive (more likely to mate with tester female when in direct competition)	*Wolbachia*		*Drosophila melanogaster* [73]
Male mating rate	Infected males mate more than uninfected counterparts	*Wolbachia*		*Drosophila melanogaster* & *D. simulans* [94]
“	Male ability to mate multiply higher in species harbouring feminizing symbiont	*Wolbachia*		Comparative analysis including 7 isopod species, five with feminizing *versus* two with CI-inducing *Wolbachia* symbionts [95]
Male fertility	Infected males do not produce mature sperm	*Wolbachia*		*Muscidifurax uniraptor* [53]
Aggregating/Lekking	Sex role reversal: females aggregate on hilltops to attract rare males	*Wolbachia*		*Acraea encedon* [22]
Female post-copulatory behaviour	Influence on offspring sex ratio via alteration of female post-copulatory position	*Wolbachia*		*Pityohyphantes phrygianus* [96]
Oviposition	Infected females aggregate offspring	*Wolbachia*		*Tetranychus urticae* [21]
“		*Wolbachia*		*Encarsia hispida* [97]
“	Cured females accept one host type at the same rate as control females but parasitized significantly fewer of the other host type.	*Cardinium*		*Encarsia pergandiella* [58]
Oviposition substrate preference	Uninfected flies preferentially lay eggs on wheat substrate, whereas infected flies do not exert apparent preference for a particular substrate		*Wolbachia*	*Drosophila melanogaster* [72]
Oviposition choice	Infection affects host choice (*i.e.*, number of eggs laid in particular host type)		*Cardinium*	*Encarsia pergandiella* [98]
**Non-reproductive**				
Larval competitive ability	Offspring of infected females are more competitive		*Wolbachia*	*Drosophila melanogaster* [20]
Dispersal	Infected females are less likely to adopt long-range dispersal behaviour (ballooning)		*Rickettsia*	*Erigone atra* [19]

In many of the cases described above a direct link is established between the infecting bacterium and the behaviour itself. Wild-type behaviour is restored by curing the host of its infection using antibiotics or by treatment at elevated temperatures. In this sense, the behavioural response is a direct result of bacterial interaction with its host and the behavioural change itself may be one of the mechanisms through which increased bacterial transmission is achieved. One illustrative example is the aggregation of eggs by spider mites [21], which is proposed to increase the chances of mating between individuals carrying compatible bacterial strains. Behavioural changes that increase the competitive ability of offspring are also likely to increase bacterial transmission relative to uninfected individuals [20]. The precise mechanisms underlying these behavioural effects on the host remain unknown but it is interesting to note that in the case of the linyphiid spider bacteria were found specifically in regions of nervous tissue associated with motor function [19].

In addition to direct influences of bacteria on the behaviour of their host there are also instances when host behaviour is altered indirectly, for example in response to manipulation of the population sex ratio. One extreme example of this is observed in a species of *Acraea* butterfly where a male killing endosymbiont results in a highly female-biased sex ratio [22]. In response to this there is sex-role reversal of lekking behaviour where females form leks around the remaining males. The case of female lekking in *Acraea* represents an extreme and rapid behavioural shift in response to a bacterial phenotype. Similarly, strongly female-biased sex ratios caused by *Wolbachia* infection in another butterfly, *Hypolimnas bolina*, lead to *increased * female mating frequency, and simultaneously to increased male fatigue associated with more prudent sperm investment per mating [23]. However there may be many more instances of subtle (and potentially transient) shifts in behaviour that might also have long-term consequences. For example, dispersal of female *Erigone atra* spiders is shown to be under the influence of a *Rickettsia* infection [19]. This is expected to result in localised perturbations of the sex ratio within the spider meta-population and as a consequence, localised variation in the strength of selection acting on traits that are sex-ratio dependent such as those involved in male-male competition or in the levels of male investment in attracting a mate.

## 3. The Evolutionary Consequences of Endosymbiont Driven Changes in Arthropod Behaviour

Perhaps the ultimate evolutionary novelty is the creation of a new species. Endosymbiont infections have been shown to be mechanisms by which gene flow can be restricted, for example through causing cytoplasmic incompatibility. Such restrictions on gene flow, even if transient or spatially restricted, may be an important component of the speciation process in particular cases [4,24]. It is unclear whether endosymbiont driven shifts in behaviour alone can also ultimately create new species although traits such as reduced dispersal e.g., as shown in the spider *Erigone atra* [19], likely reduce genetic exchange within the wider meta-population. Such effects, even if transient and/or spatially restricted, potentially contribute to the development of reproductive isolation and thus influence the broader process of adaptation and speciation.

Whilst the hypothesis that endosymbiont driven changes in behaviour can lead directly to reproductive isolation is difficult to test, it is certainly true that changes in both reproductive and non-reproductive behaviours such as mating preference or dispersal likely influence genetic diversity at a local scale. For instance restriction in the number of successfully reproducing individuals via male-killing or CI will lower the effective population size [25]. Such impacts on population size and the various impacts on reproductive traits will have further consequences for the local intensity and dynamics of sexual selection and conflict [26,27,28]. Similarly, a skewed population sex ratio will also lead to shifts in the strength and shape of sexual selection and as a consequence altered female behaviour [22,23]. Changes in mating preference may also alter the predicted linkage disequilibria amongst physically unlinked loci. Migrants canalso perturb linkage disequilibria if incoming genotypes differ from those in the resident population. These and other factors influence the ability of populations locally to adapt through natural selection in two ways. First, incoming genes or genotypes may be maladapted to the local environment. Second, changes in effective population size affect the sensitivity of local populations to the effects of random genetic drift.

Endosymbiont shifts in arthropod behaviour potentially reduce the ability of a population to adapt and persist if the outcome is reduced intraspecific variability, but in certain instances they may also favour population expansion, for instance through inducing parthenogenesis or by altering female mating rate [29]. Such rapid shifts can allow a population to expand into an empty niche. Shifts in behaviour caused by endosymbionts may also promote and maintain rather than reduce variation within a population, for instance if they increase levels of assortative mating that reduce the loss of rarer variants.

In many cases it is difficult to assess whether or not there has been sufficient time since an infection was acquired for the host and bacterium to reach equilibrium. Examples that illustrate that equilibrium has not always been reached include that of *Hypolimnas* butterflies in Polynesia, where the frequency of a male-killer decreased rapidly over a short time-frame due to the proliferation of a suppressor gene [30,31,32]. Assessing whether equilibrium has or has not been reached is sometimes further complicated by the many instances where there are co-infecting endosymbionts that may not have been acquired at the same time and that have different, potentially conflicting phenotypes where some increase host fitness and others appear detrimental [99]. Changes in host behaviour (and other host traits) that result directly from an infection or as a response to bacterial phenotypes such as sex ratio bias, are thus not only potentially transient but also rapidly changing. The magnitude of the effects that they have had at a population level may thus be difficult to establish.

## 4. Conclusions

Studies of individual species indicate that endosymbionts are a potential source of behavioural modification of their arthropod hosts either directly through their interaction with the host or indirectly as a consequence of perturbations in sex ratio. Endoymbionts may influence precopulatory reproductive isolation by altering preferences and mating rates of male and female hosts. Behavioural changes induced by endosymbionts can also potentially alter levels of genetic variation within species and the degree of population subdivision. They thereby influence the potential of a species to adapt in response to changes in the environment.
